# Causes of Exemption from Military Service

**Published:** 1865-12

**Authors:** 


### CAUSES OF EXEMPTION FROM MILITARY SERVICE.

Jno. R. Lewis, M. D., D. D. S., has compiled in the *Dental Cosmos*, some interesting statistics relating to the causes of exemptions from military service, from the Report of Surgeon J. H. Baxter, U. S. Volunteers, to the Provost Marshal Gen'l.

The total number examined in 1863 was 255,188, of these 95,134 were exempted.

The ratio of exemptions per 1000 agrees very nearly with those in Great Britain, France and Belgium. That for 1863 in this country being 314.02; in Great Britain, from April, 1842, to March, 1852, the ratio per 1000 was 345.; in France, 1831 to 1843 inclusive, 324.40. Although the aggregate ratio of exemptions are nearly the same in the different countries, yet the ratios of exemptions for certain diseases differ widely in this country from those in Great Britain and France. The exemptions on account of loss of teeth, for example, were in this country 20.49 per 1000, while in the other countries mentioned they were only from 7 to 9 per 1000. Exemptions on account of scrofula and syphilis in the United States was 5.16; in Great Britain, 28.52; in France, 10.5. (The number of exemptions in this country on account of syphilis would probably be much greater should a draft be necessary a few years hence.)

From these statistics it seems the number of exemptions in 1863 were greater than in 1864.

